# A Summary of the Claims of the Medical Art to the Consideration of the Public at the Present Day

**Published:** 1859-01

**Authors:** Theodore Mack

**Affiliations:** Lecturer on Materia Medica, Medical Department, University of Buffalo


### BUFFALO MEDICAL JOURNAL

AND

### MONTHLY REVIEW.

**VOL. 14.** JANUARY, 1859. **NO. 8.**

### ORIGINAL COMMUNICATIONS.

ART. I.—*A Summary of the Claims of the Medical Art to the consideration of the Public at the Present Day.* By THEOPHILUS MACK, M. D., Lecturer on Materia Medica, Medical Department, University of Buffalo.*

GENTLEMEN:

For the next four months we shall be engaged in the study of pursuits of the highest importance, and your future utility to society, your individual happiness, and your success in life, must, in a great measure' depend upon the doctrines you now imbibe, the habits you form, and the extent to which you avail yourselves of the opportunities for study presented to you. It shall be the constant aim and object of my colleagues and myself to insure that every facility shall be afforded to you for the acquisition of such an amount of knowledge as may render your futnre career fortunate, and add honor to the profession of medicine.

Without dilating, according to the *Pecksniffian* style, upon the ineffable feelings of pleasure experienced by the entire faculty at beholding so many zealous and intelligent applicants for initiation into the arcana of our art, allow me to present to you the claims of our profession to the public support, as they strike one living far from the turmoil of cities, and beneath the

* Introductory Lecture to the Course of 1858—59. Published by request of the Class.

strife and excitement which await celebrity and eminence. At the same time, I would draw your attention to some of the points we believe we are excelling our wise and venerated predecessors in. In the execution of this design,we, in the outset, declare that far from depreciating the labors of those who have toiled in the same vineyard during ages past, we recognize in them the great fathers of the progressive, and active interpreters of nature of the present day; and acknowledge that without their explorations, their errors, and their discoveries, we should now be found lamentably deficient. But inasmuch as the mind of man is distinguished from the intelligence of the brute by its constant advancement, each of our generations contribute to the great tower of human science, having severally received in our turn the traditions and records of the men who have played their part and left the stage, ere we were called upon to strut our hour.

I hold it to be the essential of all men of true learning, to bow to the spirits of the mighty dead, as much as it is that of quackery, where and whenever it pushes its mushroom head through the soil, to deride all that has been said or written before its ephemeral existence commenced. It is the same vain-glorious self-sufficient popinjay now, that it was a century back, and it was then the identical noisy strutting jackdaw complained of by Hippocrates more than two thousand years before. Harken to its portrait about a century ago in the great Dr. Bossy; "This renowned mountebank came to his booth erected weekly in the midst of Covent Garden market, in his chariot with a livery servant behind. His stage was erected opposite the north west collonade, Covent Garden. The platform was about sixty feet from the ground, was covered, open in front, and was ascended by a broad step-ladder. On one side was a table with a medicine chest, and surgical apparatus displayed on a table with drawers. In the centre of the stage was an armed chair in which the patient was seated; and before the doctor commenced his operations, he advanced, taking off his gold laced cocked hat and bowing right and left, began addressing the populace which crowded before his booth," &c. &c.

This was the fungus of that day; shall we raise the curtain and show a specimen of it in Yorkshire only two years since, that is to say, in our own day—we find this in a Manchester work, "The Language of the Walls." It is taken from the mouth of a quack doctor dispensing his wares on a market day in the town of Bradford. "My friends, I stand here before you independent, free and untrammeled by connections with any sect, party, profession or denomination. I thank God I am no human butcher or wholesale poisoner. I do n't come to you with bad Greek and corrosive minerals, the one to charm you, and the other to send you to your long home. No, my friends, you see these vegetables spread out before you; these are the produce of your own lovely hills, valleys, and green fields, and during the summer months many of them lend the charm of health to your meadows by their varied colors, and make the air balmy by their sweet fragrance. Not one of these, my friends, but possess a life-giving essence or a health-restoring principle. The royal poet who danced before the ark, said, that man was wonderfully and fearfully made; and his son proclaimed the everlasting truism, that man is born to trouble as the sparks fly up. (John give that lady a twopenny box of pills.) Yes, my friends, notwithstanding your liability, (another box, John,) to disease and death, (Did you say a two-penny box, Sir? another box, John,) here is a safe and speedy remedy, (attend to that gentleman, John,) for every disease mortal flesh is heir to. My friends, you do well to supply yourselves while I am here, (two penny boxes, John.) During the course of the ensuing month I am obliged, by previous arrangement, to visit the following towns, (two-penny box, John): To-morrow morning I leave here for Sheffield, (attend to the lady with the child in her arms, John,) and from there to Chesterfield, (Cure headache, did you say? why, my dear fellow, one box would cure a horse's head, which is four times as large as yours); then on to Derby, (give the child two pills at bedtime, and continue the dose for a month,) (ah! poor man, your swimming in the head arises from long hours of labor at a sedentary employment,) (two large boxes, John); then I go to Hexham, Halt Whistle, Brampton, and Carlisle, (My friend, your anatomical man-killers would transport me if they had the power; thank God I hold my authority from a higher power than that which deputes them to poison, and kill by the knife); then I go to Penrith, Shap-Kendal and Manchester." This is a specimen of their logic; this is a type, a little broad, of our own gentry, who lecture on physiology gratis, and may be consulted afterward for a consideration, or propound, and expound to gaping audiences new systems of infallible cure, understood and practised best of course by the learned orator himself, and such are generally the spirits who have so sublime a contempt for the writings and opinions of the ancients. Montaigne after praying "God grant that one day medicine may bring me some good and perceptible succor," bitterly said, "The arts which promise to keep our body in health, and our souls in safety, promise much; nevertheless none fulfill their promises less." This was a growl from an erudite and eminent grumbler, then in agony from a disease incurable at the time, but now daily and certainly cured by the knife in the hands of the surgeon, viz., stone in the bladder, and often, as as we are upbraided with the uncertainty and inadequacy of medicine by supercilious faineants or sophists, read up in the literature of the days of our infancy, we safely hold, that except in vocations wholly resting upon mathematics, we not only are not surpassed, but are not equaled in any other walk of life, both in the certainty of our opinions and in the blessings we confer upon society.

Where do you point out greater uniformity of sentiment? Is it in the church? where not only are the parties and sects so different in forms of faith, in external rites and internal policy, but the learned doctors of each denomination are continually at issue among themselves concerning interpretations and theories. Is it then in the management of the ship of state? Every election teaches us how the future puzzles the most astute politicians; every session of legislature leaves behind it the traces of fatal blunders; and how many political economists are of accord upon such subjects as the rights of citizens, protective tariffs, free trade, education, municipalities, public works, and commerce.

The most consummate skill in the counting-house we see constantly rewarded with want and degradation, often involving, in the same ruin, hundreds of dependants and friends.

The wisdom and foresight of the most able engineers and navigators, as well as the most scientific men of two of the mightiest and most enlightened nations of the earth, could not prevent the snapping of the Atlantic cable. Alas! what would become of the poor patient, over whom so many disputations, experiments and theories had been exhausted as over this same 2,000 miles of wires. His fate, I fear, would have been inevitably that of the *Malade* under the care of M. Tomès, in Molière's play of "L'Amour Médecin." This uncompromising son of Æsculapius informs us that although the disease was pressing, and the friends in the highest distress, he held on to his opinion sternly against three others in consultation; "and the patient died bravely during the dispute."

Look at the state of agriculture — the primitive, the earliest studied, at all events, the most experimented of human arts; how many disagree upon the nature and application of manures; how few agree as to the causes and remedies of the various insects, fungi and diseases which attack our cereals, and impoverish the country in sections.

Is it, then, to that profession which has given to us a Mansfield, a Hale, a Kent and a Webster, that we are to turn for unity and accuracy? If our enemies cast in our teeth the proverbial uncertainty of physic, what will they say to "the glorious uncertainty of law?"

Without extending our comparisons, there is evidently just as much hypothesis in all other professions and branches of knowledge as in ours.

The renowned English historian, Macauley, expressly states: "The term of human life has been greatly lengthened in the whole kingdom. In 1685, not a sickly year, one in twenty of the inhabitants died, while at present only one in forty dies. The difference between London in the 17th and London in the 19th century, is as great as between London in ordinary years and London in the cholera."

Let it not be deemed arrogance to ascribe almost wholly to the labors of my own profession this wonderful amelioration. Surely, there are few so obtuse as for one moment to entertain the thought that I mean to refer this marked change to the administration of drugs alone. No; this I regard as but the minor portion of the physician's duties. It is by continual, earnest interrogation of nature, and observation of her laws, that he is able to indicate when we transgress; to counsel not only a patient, but a nation; to meet or avert the impending epidemic, or, when the malady has actually made an invasion, to place in a proper position to sustain its attacks, districts, cities, towns, families, and individuals, with the least wear and tear to the system; and finally, to suggest the remedies to dislodge the foe when he has established a foothold.

Those exertions for sanitary reform and the prevention of the adulteration of food, in which medical men have been so preëminent, are also productive of high moral benefits to society; for it is an indubitable verity, that causes which lower the tone of the system produce mental apathy, indolence, and cravings for spirits, ending in drunkenness, profligacy and poverty. Thus the poor laborer and other children of toil, victims of miasma, malaria, filth, and insufficient or bad food, their wives and children, stricken by disease, go down from one stage of depravity to another, till the intellectual faculties are rendered torpid; moral feeling, religious obligations and domestic ties are destroyed; all respect for law or property is abandoned; ultimately the prospect of the future becomes desperate, and hope is withered to be supplanted by despondency and recklessness.

The pestilence so often adduced as one over which medical science has no control, — the Asiatic cholera, — by prudent sanitary regulations and other precautions emanating from our researches, and published by members of our profession, or imparted by them to the people, has been mitigated. We have, in fact, already divested this appalling scourge of at least half its terrors.

There are still in our midst many aged persons who can look back with horror at the dismay and confusion following the appearance of small-pox in any neighborhood half a century ago. Does it create any such commotion now? or do we meet its victims in every thoroughfare as once we did, — who have been only too glad to compound for life with the loss of every trace of a beauty which once delighted the eyes of those who were dear to them.

Cromwell died of ague, and shrunk from Jesuits' bark as too Papistical for his Protestant stomach. Who need die of ague now?

Consumption was defiantly instanced as a disease in which all remedial measures were utterly futile; but we find now that life may be prolonged, even in this subtle distemper, for periods of three to ten years, and occasionally we can record examples of perfect cure.

The knife of the operator no more chills the blood with horror. The applicant for surgical aid may undergo dismemberment, while dreaming of pleasant scenes long forgotten in memory's sunniest days, or babbling of the green fields of jocund childhood.

The beautifully simple theory of the excito-motory nervous system, fully and satisfactorily borne out by the discoveries of the day, has been most important in its applications to pathology, and is becoming more and more valuable in its practical bearing upon the treatment of disease. By it, irritations are reflected from one part to another. The enemy's first success at an out-post is speedily felt at the citadel; or the derangement of a distant dependency points to something wrong at the seat of government. By reference to this chart of our sensations, we are generally enabled to trace diseases to sources which must otherwise remain unsuspected.

It is no longer, as in the days of Sganarelle, "The blunders are not for us, and it is always his own fault who dies." Even at that time it was *un médecin malgné lui*, whom Moliere makes, to say, "In fact the beauty of this profession is, that among the dead there exists a civility, a discretion, the greatest in the world, and we never meet one who complains of the doctor that killed him."

Our knowledge of the nature, causes and treatment of the many-headed hydra, FEVER, has become singularly clear and successful; and chemistry, in leading us back to the recognition of the truth of a limited humoral pathology, has forced us to retrograde one step only to advance many strides. In the distinction also of continued and typhus fevers, as marked by an eruption, from another very similar fever, the typhoid, who will deny that we have attained a decided advantage?

The diagnosis of Bright's disease alone, another modern discovery, has reduced the treatment of a vast assemblage of puzzling symptoms to a scientific basis.

Surgery we find marching onward hand in hand with her sister sciences. It is estimated that the saving of life in surgical cases exceeds the results at the commencement of the present century by more than 35 per cent. And then, in that branch of our art whose province it is to aid the travail, and afford ease to the numerous ills of that moiety of our race, who alone can make a garden of this wilderness of life, our statistics evince that the labors of those zealous men who have devoted their energies to this department have been crowned with the most flattering returns, and our well-directed attentions are now rewarded in our daily visits, by grateful smiles and cordial greetings, instead of encountering a brow frowning with pain, or the querulous complaint of unalleviated distress.

Auscultation and percussion have acquired a scientific exactness of the most surprising nature. Not only have these two means of exploring the hidden "causes in the bud" given an exquisite precision in discernment of diseases of the chest, but they have brought to light others wrapt formerly in inscrutable mystery.

The mortality in the Parisian hospitals shows a saving annually of five hundred beings, beyond what were saved fifty years ago, and further, the period of suffering for patients admitted has been curtailed one-third.

In many other special diseases besides those we have alluded to, the improvement is truly surprising. In one of those where one man died formerly out of every fifty-six affected, at the present day only one out of 300 dies.

I am only speaking to you of the tangible salient benefits conferred by the vocation upon the community, else might I not dilate upon the astounding revelations of the achromatic lens. One thing is indisputable, that the microscope has placed many affections completely in our power, in the treatment of which, until quite recently, we were perplexed with hypothesis and doubt.

One hundred and fifty years ago, maternity cost one life in forty, and now, we are confident in the assertion that not one life is lost in 250 cases.

We have cited a powerful authority in proof of the increased longevity in England. An equally reliable one states, that in France, notwithstanding the many violent deaths consequent upon years of political struggling, the duration of life has been increasing, equal to fifty-two days a year.

Contrast the interior of a hospital of our own epoch, and here in your own city, with that of the best European institution of the same nature eighty years since. The scrupulous cleanliness; the disinfecting agents; the scientific ventilation and heating; the elegant fracture apparatus, where anatomy and mechanics combine to achieve all that is possible for the sufferer, both as to present comfort and final result; the simple water-dressing for the open sore; the wound maintained in apposition, to favor the healing process by the first intention; compare all this with the condition of the old hospital, crowded apartments, bad air, filthy persons, clumsy mechanical expedients of every kind, rancid unguents, fœtid ulcerated mouths, filthy poultices, nature-baulked and simple incisions kept forcibly open. Survey all this, and then dare any man tell me that "medicine as a science stands still while all the others advance."

Our remedies, by a proper application of the method of the immortal Lord Bacon, are hourly undergoing a rigid examination, and we are fast consigning to merited oblivion an endless category of worthless incumbrances. An actual bona fide knowledge of the benefits of different modes of cure is gaining ground, which by concentrating and extracting the chemical agents, to which medicines owe their activity, we are making them more certain and easier of control; and lastly, vast improvements are being effected in their form and mode of exhibition. Now all this beneficial innovation' and time would fail me to adduce to you how much more has been chiefly brought about by individual exertions, by patient, honest collation of fact and wise inductive reasoning therefrom.

Inductive science takes her stand upon the practical ground of every sane transaction; she sets up her axioms upon that bond which connects facts that have been, with facts that must follow. This mode of acquiring knowledge is instinctive in the dawn of intelligence; but as mind becomes more developed, it becomes involved in many intricacies, which, as reason enlarges her sphere of vision, it becomes her business to unravel, analyzing the com plicated web of phenomena into a system from which we establish what are called "the laws of nature." To do this, well recorded facts must be rigorously compared ere they are discussed, with a view of declaring relations, and methodically proceeding to the appreciation of causes.

The difficulties attending this only true mode of advancing science may be conceived, when I tell you that fourteen centuries of close observation has barely sufficed to the establishment of one great fact, the inestimable discovery of Harvey, the circulation of the blood. It appears a marvellous lapse of time for eliciting that which, now that we know it, we are only puzzled to comprehend how it was ever unknown; but we must take into account the servility of the human mind to old established opinions, the blindness with which prejudice afflicts even talented men, so that they continue acquiescent in errors so baseless that they vanish the moment they are challenged. Men, not brought up to the perseverance and nicety of observation, can hardly understand how obvious and simple truths remain so long overlooked, and more, the rare endowments of independence of mind, impartiality and liberality to the observations of others, which constitute a *good observer*, are too seldom found among those otherwise gifted in this delicate art.

I sincerely hope that none of you ever have experienced *hunger* in its dreadful reality; and I believe, gentlemen, that a discerning public will never allow an alumnus of this college to suffer from the pangs of starvation; yet there are few of us who have not, at some time of life, particularly in the morning of our days, endured the stimulus of appetite a little sharper than quite agreeable; yet will you tell me the proximate cause of this sensation? If you do, I will guarantee that your name shall be enrolled among the *savants* of your day. Methinks I hear some embryo Harvey exclaim— "Hunger! why hunger is from want of food," and lo! the gordian knot is cut. Absence of nourishment may be the *primary*, but is not the *proximate* cause. It is not caused by the friction of the walls of the stomach when empty, nor by the gastric juice accumulating and attacking its coats; nor by the distention of the little tubes leading from the glands, which secrete the gastric juice: probably this sensation is due "to the general state of the system when in want of nutriment, and that the stomach is the seat of the sensation, just as the drooping eyelid manifests the condition which demands sleep," but the exact origin of this familiar sensation has never been clearly demonstrated.

The fact is, that in a science like medicine, this method of inquiry is unfortunately not the most natural and suggestive. Under circumstances of excessive complication in a department of investigation, in which success leads to celebrity and sometimes to fortune, the result of a single experiment, the striking issue of a novel process makes its way at once to the inductive instinct without being subjected to the scrutiny of reason. The comparison of multitude with multitude, the destruction of errors by mutual collision, and the slow emergence of truth from the conflict, by its outstanding vitality, belong to a maturer age than that in which medicine had its origin, or attained its present importance.

Notwithstanding these obstacles, extraordinary progress has been made in the last half century, by the application of numerical methods, and averages to the history of disease. Averages may in some sort be termed the mathematics of medical science, and their success gives assurance already of the results that may hereafter be expected from the same source. Through medical statistics lies the most secure path into the philosophy of medicine.

As exemplifying the progress made by inductive philosophy in our art, we may mention some of the facts in the study of food, lately submitted to the public eye, — a line of research marked by many triumphs. That green sward so grateful to the eye after the snow and leafless inanity of dreary winter have passed away before the genial warmth of spring, not only renders your footstep more luxurious than the costliest carpet woven by the hand, but when the sensation we just alluded to leads you to turn your face homeward, you will find it smoking on your spit in a form fitted for your nourishment. For, you must know, that the same constituents, — the albumen, casein and fibrin, that supply first the wasting of the tissues in the ox, and then performs the same kindly office for you, — are found in the herb of the field, requiring a modification almost inappreciable in the laboratory, to make flesh and muscle in the man; in fact the ox eats the grass and we eat the ox, having prepared that salad in a proper way. A head of cabbage will prove an Apician morsel to the rabbit, but a dog would prefer a bone of the rabbit to the finest specimen of that vegetable he ever saw. The identity of these protein compounds has advanced our knowledge of what goes on in the inner man to a surpassing extent. The nutritious power in wheat is found to be gluten, and after isolating and examining these components of food, it is found that not one of them singly can support life, but a due admixture of them all in just proportions; and what may surprise some of you, it has also been demonstrated that health cannot be maintained without iron, which is an essential ingredient in the blood corpuscles; and further, like lucifer matches, phosphorus and sulphur are indispensable to our composition.

These are a few of the facts in relation to alimentation, which, of course, I only make allusion to at present to give a glimpse of the nature of our labors in physiology and animal chemistry. Ere we shake hands at the close of the session, I hope each of you will be competent to show the tendency of those truths, and explain the ratiocination springing out of these and its utilitarian applications.

And now, gentlemen, in return for a life of study, for enriching their fellow-citizens by valuable discoveries, and in recompense for dwelling in the midst of pain of sickness and death, the dearest reward to every physician who truly loves his noble art and duly appreciates its lofty duties, would be that the people should enforce that "no one shall be allowed to treat diseases, whether he calls himself allopath, homœopath, isopath, physiopath, eclectic, botanic, or by any name, until he has shown before a proper tribunal that he has made the organism of man and his diseases a special study."*

Anciently, in some states, high privileges and immunities were awarded to the practitioners of medicine, They seek not now any such distinctions; they simply demand, as a just appreciation of their liberal art, that mankind will take care of themselves, by ceasing to encourage a banditti of swindlers, ever on the alert to chisel their dupes out of life and good health, and last, not least for them, the means of supporting both.

In conclusion, suffer me to impress upon you the paramount importance of rigidly economizing and improving every moment of your *séjour* here. The golden hours now squandered can never be made up for in after life; and your prestige at a future day must depend upon your evincing at your *début* that you are men of learning, while no opportunity will, in days to come, offer for the improvement that you are now susceptible of, ere the sober realities of life engross all your energies. By careful attention to the lectures, you will be able to perceive what will prove most to your advantage in reading, and much will be rendered plain and intelligible, which in reading, unaided by oral instruction, might seem obscure and indefinite; but remember, that it is from books you must study any subject systematically. Methodical habits are all-important throughout the entire course of your studentship; "Order is Heaven's first law," and for the lack of it I have too often seen many a fine genius drifting, the sport and buffet of unruly, but frequently brilliant conceptions.

"Studium sine calamo, somnium." Your pen or pencil should be ever ready to note, when in the college or at your desk. Your conversation should always be made to turn upon some topic connected with the objects which have collected you together within these walls. If your communications with your fellows be thus prudent, no unbidden guest will intrude upon your moments of reflection. If your studies be confined to professional subjects, your conversation chaste, refined, and having mutual improvement for its aim, rely upon it, your mind, when alone, will be constantly engaged in the contemplation of profitable things.

Having thus foreshadowed what we expect from you, and what we claim

---

*Comegys.

to be able to impart to you, we will jointly sally forth into the domain of science, and time, I trust, will prove the fragrance and choice of the bouquets we shall cull along her flowery paths.

